# Adult Intestinal Malrotation in a Non-Paediatric Hospital in Trinidad: A Case Report and Literature Review

**DOI:** 10.7759/cureus.12305

**Published:** 2020-12-26

**Authors:** Johnathan K Jarvis, Amrit Rambhajan

**Affiliations:** 1 General Surgery, General Hospital Port of Spain, Port of Spain, TTO

**Keywords:** adult intestinal malrotation, ladd, malrotation, trinidad and tobago, trinidad, caribbean, whirlpool sign, stringer, midgut rotation

## Abstract

Intestinal malrotation (IM) is a congenital aberrancy of midgut rotation during development, which manifests among neonates more than adults. Older reports have estimated an incidence of one in 6,000 live births, which is now as high as one in 500. This congenital anomaly is generally indolent in the adult population. Recent literature research has failed to reveal any publications regarding the incidence within a Caribbean population. This paper aims to discuss the isolated case of a patient with this rare condition, who presented to a non-paediatric centre in Trinidad. This case highlights the implications of the initial radiological interpretation in conjunction with perioperative and intraoperative decision making.

An 18-year-old male presented with a one-day history of abdominal pain, radiating to the epigastrium with nausea and excessive vomiting. Vital signs and blood investigations were normal. Initial CT scan results were interpreted as an internal hernia, which was surgically managed as such. Repeat imaging and a second laparotomy were required to correctly diagnose and perform the appropriate Ladd procedure.

IM occurs due to the arrest of rotation of the midgut during fetal maturation. The incomplete rotation variant was seen in this case and is predominantly responsible for the symptomatology and morbidity associated with adult intestinal malrotation (AIM). Stringer has classified these anomalies based on the stage of embryonic development that is disrupted. CT helps with diagnostics in 97.5% of cases. This case highlights the implication of incorrect assessment on imaging and how it may misguide the interpretation of the findings at laparotomy leading to inappropriate surgical procedures. As many as 20% of cases undergo surgery without adult intussusception diagnosed.

The incidence of IM seems to have increased but is scarcely encountered in the adult setting. When encountering this condition at a low-volume centre in the Caribbean, the adult specialist may be blindsided, and unknowingly underprepared without a high index of suspicion. Diagnosis at childhood should be discussed with the family and again with the patient on approaching adulthood. ﻿Patient education may help with the surgical assessment.

## Introduction

Intestinal malrotation (IM) is a congenital aberrancy of midgut rotation during development, which manifests among neonates or infants more than adults [1,2]. Older reports have estimated an incidence of one in 6,000 live births, which is now reportedly as high as one in 500 [3,4]. The increase in incidence may reflect advancement in imaging modalities and heightened vigilance to screen for this condition.

A paediatric database analysis of 2,744 cases has found that 60% of patients presented before the age of one year and 75% by the age of five years [5]. In contrast, another single-centre study that reviewed 170 cases over more than a decade found that 48% of cases occurred in people who are over 18 years of age [6].

The assumption that the presentation of IM is limited to the paediatric population may reveal itself as a shortfall to the adult specialist. The estimated incidence of adult intestinal malrotation (AIM) is 0.2% [7]. This congenital anomaly is generally asymptomatic or clinically indolent in adults. The patients usually show delays in seeking medical attention and they often show up with a history of more than six months, with 10-15% of AIM cases presenting with the critical condition of an acute midgut volvulus [8,9].

Recent literature research has failed to reveal any publications regarding the specific incidence within a Caribbean population. A study by Trevor et al. reviewed the incidence of congenital intestinal obstruction at a paediatric centre in Trinidad and reported an incidence rate of 3.14 per 10,000 live births [10]. It may be extrapolated from this that symptomatic IM may be arguably less prevalent. Over a period of seven years, they also reported one case of obstructing IM resulting in perforation [11].

In 1992, the aforementioned hospital reported a total of 51 cases under the age of 10 years [12]. Though the time of data collection and sample size were not readily accessible to further build on this finding, we feel this finding to be significant given that the region in reference was the same as in our case.

This paper aims to discuss the isolated case of a patient with this rare condition, who presented to a non-paediatric, low-volume centre in Trinidad. This report highlights the implications of the initial radiological assessment and interpretation in conjunction with perioperative and intraoperative decision making.

## Case presentation

An 18-year-old Afro-Trinidadian male presented with gradual-onset central abdominal colicky pain, radiating to the epigastrium for one day. The patient had experienced associated nausea and three episodes of large volume bilious vomiting with undigested food. He complained of malaise but remained afebrile. His vital signs were normal on examination. His blood pressure was 126/94 mmHg; his heart rate was 15 beats per minute and his respiratory rate was 83 breaths per minute. The abdomen was soft, with mild tenderness in the periumbilical region and right upper quadrant of the abdomen. There were no signs of peritonitis and nor anything to suggest typical appendicitis. The digital rectal exam revealed normal stool devoid of blood or anorectal masses.

The complete blood count was within normal parameters [Haemoglobin (Hb): 14.5g/dL, white blood cells (WBC): 8x10^3^uL]. Renal and liver function tests were within normal ranges. Plain supine abdominal radiographs demonstrated distended large bowel but two air-fluid fluids on erect films.

**Figure 1:**
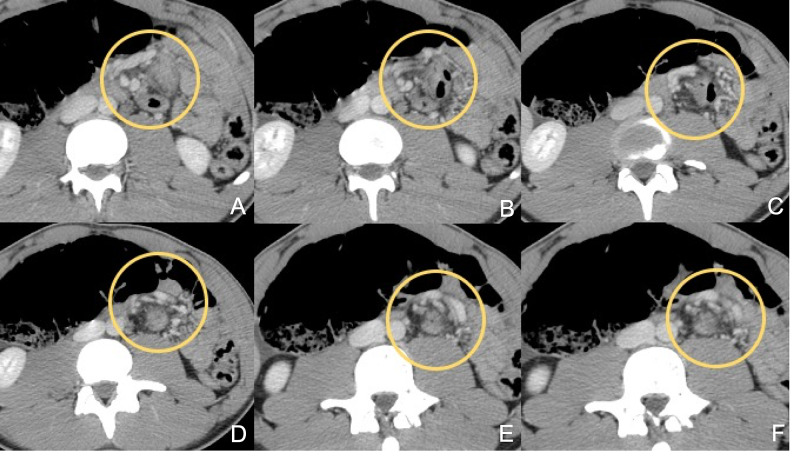
CECT view 1 Progressive axial slices showing the ‘whirlpool’ sign (yellow circle) CECT: contrast-enhanced computed tomography

**Figure 2:**
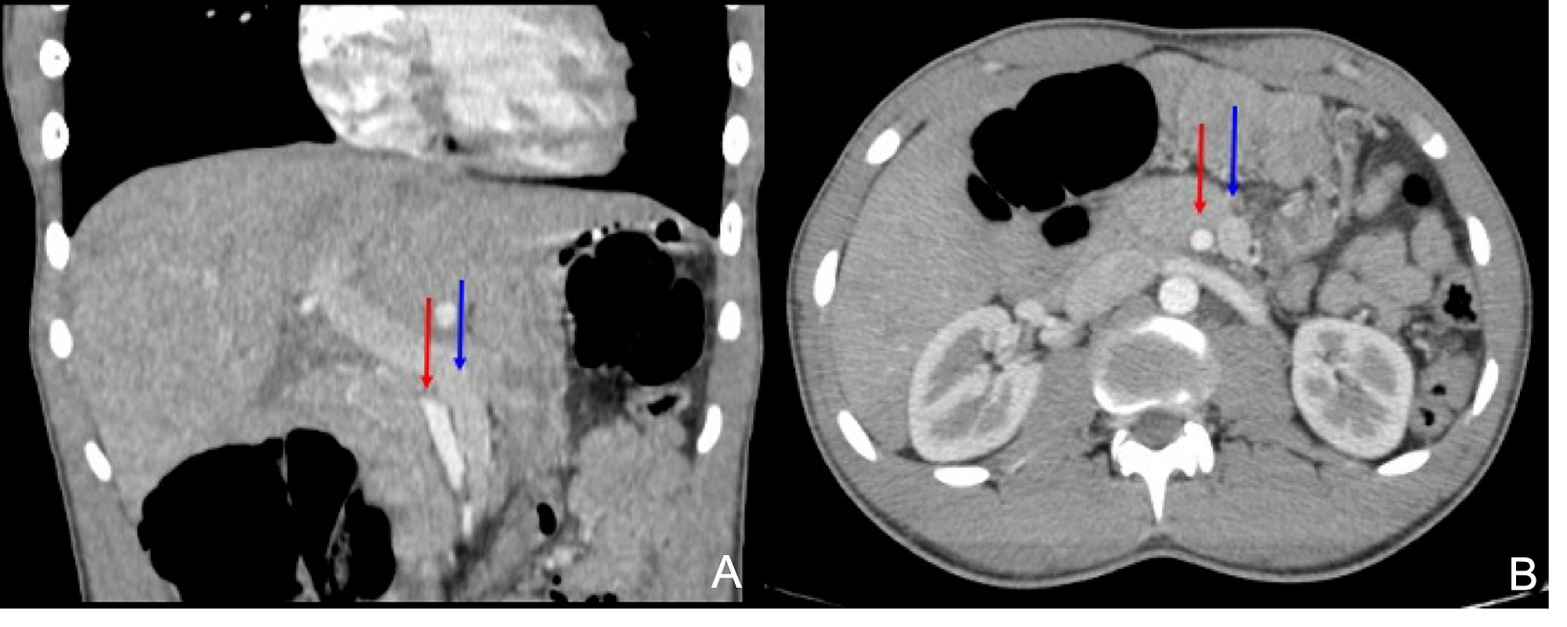
CECT view 2 Coronal (A) and axial (B) views demonstrating 'reversed positioning' of the SMA (red arrow) and SMV (blue arrow) to the left CECT: contrast-enhanced computed tomography; SMA: superior mesenteric artery; SMV: superior mesenteric vein

He was further investigated via contrast-enhanced CT (CECT). This revealed the ‘whirlpool’ sign (WS) (Figure 1), as well as the reversed relative positioning of the superior mesenteric artery (SMA) to the right of the superior mesenteric vein (SMV) (Figure 2). This was initially interpreted as a possible internal hernia by the radiologist on call.

**Figure 3:**
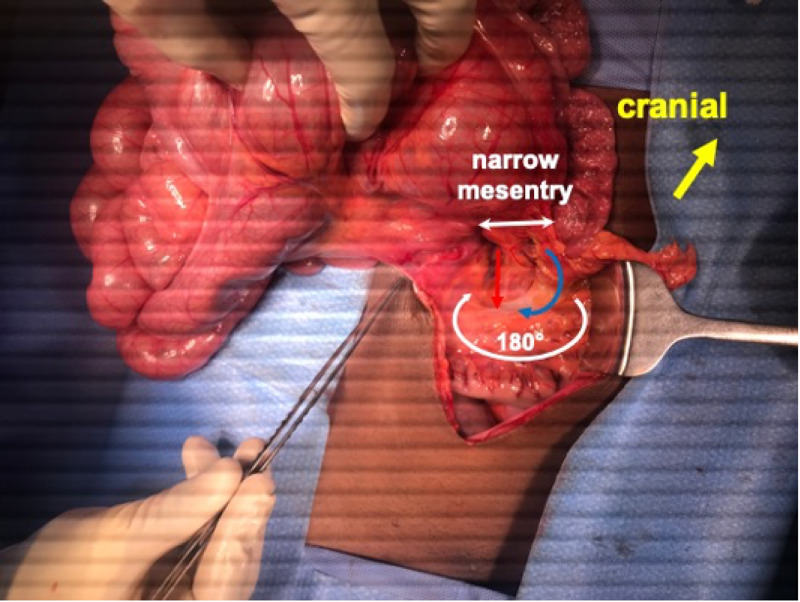
Intraoperative findings showing the narrow superior mesentery with 180 degrees of clockwise rotation SMV (blue arrow) located to the left of the SMA (red arrow) SMV: superior mesenteric vein; SMA: superior mesenteric artery

Following adequate resuscitation, the patient was taken to the surgery by a senior resident and an attending with over 10 years of experience. Laparoscopy was considered; however, instrument availability was a limiting factor in our setting. A midline laparotomy skin incision was performed. On initial examination, the caecum was instantly noted in an ectopic position in the right upper quadrant. Further assessment while following the small intestine revealed a shortened small bowel mesentery with 180-degree clockwise rotation (Figure 3).

This would have been attributed to the SMV appearing to the left of the SMA on the previous contrast studies. The bowel appeared viable as there was no evidence of ischaemia. Careful assessment of the positioning of the mesenteric vessels and gentle anticlockwise detorsion was achieved.

**Figure 4:**
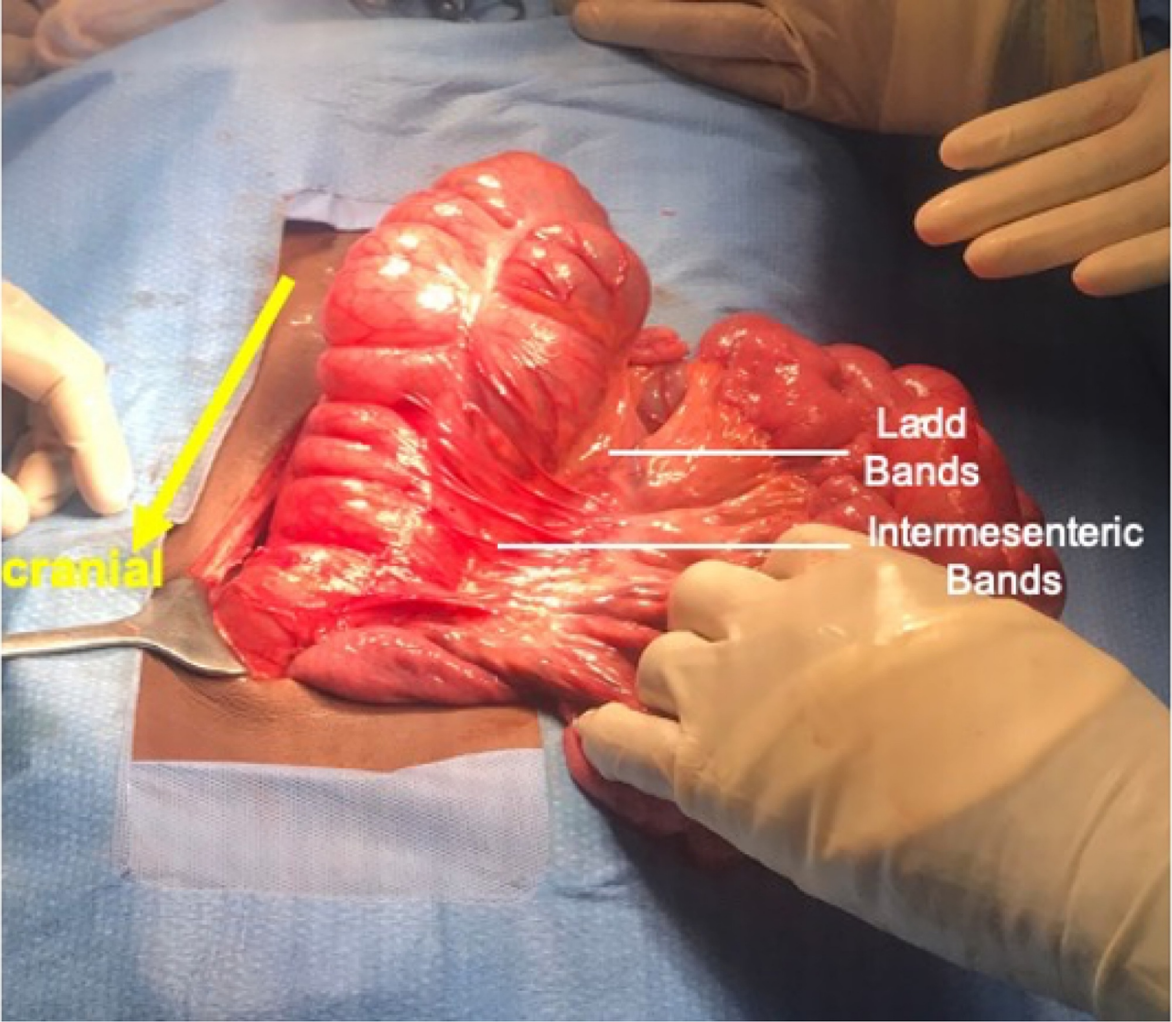
Intermesenteric bands between ascending colon and small bowel mesentery Ladd’s bands are also seen (deep to intermesenteric bands)

Congenital intermesenteric adhesions were noted between the ascending colon and small bowel (Figure 4). Adhesiolysis was performed to facilitate repositioning of the bowel. The appendix was normal; however, an appendectomy was completed to avoid any future diagnostic dilemma. A cecopexy was performed attempting to reposition the caecum in the standard right lower quadrant while placing the small bowel medial to the ascending colon, thereby concluding the operation.

**Figure 5:**
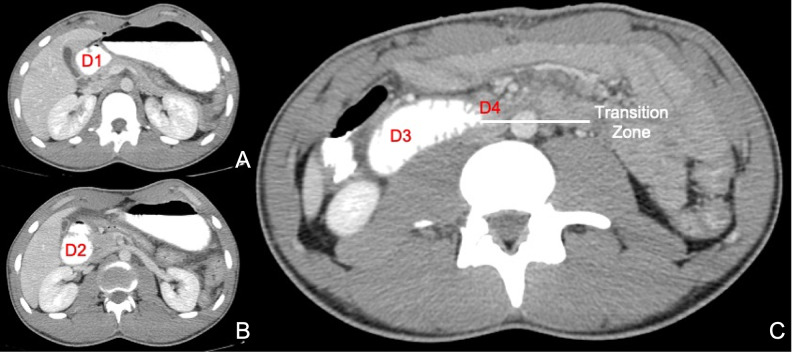
Postoperative CECT Axial views showing pooling of contrast up to D2 (A, B) and compression of D3 with a transition zone between D3 and D4 (C) D1: first part of duodenum; D2: second part of duodenum; D3: third part of duodenum; D4: fourth part of duodenum; CECT: contrast-enhanced computed tomography

The patient had three uneventful postoperative days. On the fourth postoperative day, the patient experienced sudden-onset projectile vomiting. A nasogastric tube (NGT) was positioned, draining over a litre of bilious effluent on placement. Oral contrast was administered followed by a CECT of the abdomen showing pooling of contrast in the stomach and compression of D3 with little contrast beyond this transition point (Figure 5).

The preoperative and postoperative CT scans were compared and re-examined in conjunction with the findings at the laparotomy to diagnose a Stringer IIIa malrotation. The patient returned to the theatre for re-exploration. The cecopexy was reversed. Complete adhesiolysis of Ladd’s bands followed by Kocherization was conducted. The bowel was then arranged in a nonrotation position with the small bowel to the right of the midline and the colon to the left.

Intraoperatively, the patency of the small bowel was assessed by instilling water via the NGT while occluding the mid jejunum. Distension of bowel beyond the duodenum was observed confirming the passage of contents beyond the initial transition point.

The patient began oral fluids the next day and tolerated a normal diet by day three without any associated abdominal discomfort. He was discharged from the hospital on day four with no further issues.

## Discussion

IM is an anatomical anomaly caused by the failure or arrest of rotation of the midgut during fetal maturation between the third and tenth week of development [3]. Ordinarily, the midgut and proximal large intestine elongate, coupled with 270 degrees of anticlockwise rotation along the superior mesenteric arterial axis [3,13]. This results in the regular position of the ileocecal junction in the right iliac fossa. The duodenum is set posterior to the superior mesenteric vessels and to the right of the midline in the upper quadrant and the transverse colon resting anteriorly [3,13]. This normal orientation carries a long wide-based mesentery.

Interruption in this process results in nonrotation or incomplete rotation anomalies. The nonrotation variant is the one that is frequently encountered. The small bowel remains situated on the right of the abdomen with the colon contralateral [14]. Most often this alignment allows the patient to remain asymptomatic and most are diagnosed incidentally on radiography or at laparotomy while evaluating another disease process [14].

Incomplete rotation, as in this case, occurs when there is an arrest during the revolution, usually at 180 degrees. The pre-arterial duodenojejunal limb remains to the right of the midline with the post-arterial distal segment and caecum located in the upper mid-abdomen [13,14]. This variant is predominantly responsible for the symptomatology and morbidity associated with IM. During the third stage or zygosis of the proximal large intestine to the lateral peritoneum, the abnormal position of the caecum becomes fixed by fibrous bands called Ladd’s bands, which traverse the duodenum [14]. These bands may cause an extrinsic compression on the duodenum causing proximal intestinal obstructive symptoms [3,14]. Secondly, there is an increased risk of a midgut volvulus as the base of the mesentery is narrow [14].

Infrequent variants include hyper-rotation beyond 270 degrees and reverse rotation, where the duodenum lies anterior to the SMA and right colon creating a paraduodenal hernia [3]. Stringer went on to classify these anomalies based on the embryonic stage of development that was interrupted (Table 1) [15].

**Table 1:** Stringer’s classification of intestinal malrotation* *[15] SMA: superior mesenteric artery

Type	Embryonic stage		Description
I (nonrotation)	<6th intrauterine week		Nonrotation of colon and duodenum before
II (duodenal malrotation)	6th–10th intrauterine week	A	Duodenal nonrotation with normal colonic rotation often results in the formation of Ladd's bands
		B	Reverse rotation of both duodenum (anteriorly to SMA) and colon (posteriorly to the SMA)
		C	Reverse rotation of duodenum only (entrapment of small bowel)
III (duodenal and caecal malrotation)	>10th intrauterine week	A	Duodenum to the right of midline and caecum lies high predisposing to volvulus
		B	Normal rotation of duodenum, incomplete fixation of the hepatic flexure often with Ladd's bands
		C	Incomplete attachment of caecum and mesocaecum (mobile caecum)
		D	Paraduodenal hernias near the ligament of Treitz due to variable fixation (pseudo-malrotation)

The clinical presentation of AIM includes three types: incidental, chronic, and acute [16]. Chronic presentations are subtle, and symptoms are vague and often nonspecific, protracting over months or years. Colicky abdominal pain, bloating, nausea/vomiting, constipation, and food intolerance, and weight loss, from remitting episodes of midgut volvulus or duodenal compression are the usual symptoms [16].

Frequent symptoms during the acute presentation are severe epigastric or periumbilical pain, vomiting, and distension. The presence of peritonism, haematemesis, haematochezia, or shock implies perforation or vascular compromise with possible necrosis, which indicates emergent laparotomy [14].

A recent systematic literature review by Neville et al. found that CT helps with diagnostics in 97.5% of cases, regardless of contrast enhancement [7]. The most commonly observed characteristics are as follows: (1) abnormality of the SMV/SMA relationship (58%) i.e. the SMV lies to the left or anterior to the SMA; (2) midgut volvulus (50.6%); (3) the ‘whirlpool’ sign (30.9%) i.e. twisting of the mesenteric vessels; (4) small bowel obstruction (27.2%); and (5) a right-sided duodenal-jejunal segment (17.3%) [7].

This case echoed the radiological findings that were expected; however, a closed-loop obstruction from a possible internal hernia was the diagnosis initially entertained. The implication of this assumption was mirrored in the findings interpreted at the initial laparotomy and the incomplete surgery performed. The literature maintains that 20% of cases of AIM underwent previous abdominal surgery without diagnosis [7]. This may reflect encounters of this rare entity by less experienced surgeons.

Though ultrasound is not recommended for diagnosing malrotation and reportedly has a sensitivity of 62.5%, it is appropriate for children and infants. The gold standard remains an upper gastrointestinal series that depicts the following: a displaced duodenum with the ligament of Treitz to the right, a ‘corkscrew’ duodenum sign, or a ‘beaked’ appearance of the proximal jejunum if a volvulus is present [16,17]. Patients in the acute setting should undergo resuscitation, nasogastric decompression, and take empirical broad-spectrum antibiotics preoperatively, given the threat of bowel ischaemia and subsequent necrosis [18]. Following this, the approach should be to proceed directly to laparotomy.

The recommended surgical procedure was modified after William Ladd in 1932, who first described the division of the fibrous bands involving the duodenum, caecum, and right lateral wall [16]. The Ladd procedure consists of five key nodal points. Firstly, any torsion of the bowel must be untwisted (counterclockwise) and assessed for ischaemia. Indisputable necrotic segments warrant resection. Secondly, adhesiolysis of the Ladd’s bands to facilitate Kocherization and straightening of the duodenum should be performed. Further division of intermesenteric bands expands the base of the mesentery. Appendectomy avoids future diagnostic dilemmas with an ectopic appendix. Lastly, the bowel is returned to its natural, nonrotated orientation. Suture pexy is not recommended [16,19].

The steps taken in the prime operation predisposed the patient to re-obstruction. Failure to divide the Ladd’s bands permitted extrinsic compression of the duodenum, compounded by fixing of the caecum in an unnatural orientation in this patient. Thus, a poor outcome can be seen associated with the omission of any step of the Ladd procedure.

Insufficient evidence exists in favour of elective Ladd procedures for asymptomatic adult patients, though it is proposed by a large number of experts, to avoid possible future complications [20]. In the absence of an acute volvulus, the laparoscopic approach has demonstrated to be as effective as an open approach, with the added benefit of a minimally invasive surgery [19].

## Conclusions

The incidence of IM is seemingly on the rise but remains scarcely encountered in the adult setting. When encountering this condition at a low-volume centre, the adult specialist may easily be blindsided, being unknowingly unprepared to perform the correct Ladd procedure. It is self-evident that irregularities detected on imaging in the acute virgin abdomen should encourage the pursuit of other subtle congenital anomalies that may impact surgical decisions. It would be prudent that any incidental diagnosis made during childhood should be discussed with the family and again with the patient on approaching adulthood. ﻿They should be educated on their condition, and on the signs and symptoms to prompt urgent surgical assessment, should observation be implemented.
